# Aortic stenosis: a review on acquired pathogenesis and ominous combination with diabetes mellitus

**DOI:** 10.1186/s43044-023-00345-6

**Published:** 2023-04-07

**Authors:** Pranay Wal, Shruti Rathore, Namra Aziz, Yash Kumar Singh, Arpit Gupta

**Affiliations:** 1 Pranveer Singh Institute of Technology (Pharmacy), Bhauti, Kanpur, UP 209305 India; 2 LCIT School of Pharmacy, Bilaspur, Chhattisgarh 495220 India

**Keywords:** Aortic stenosis, Diabetic mellitus, Valve calcification, Calcified aortic valve stenosis, Degenerative aortic stenosis

## Abstract

**Background:**

Aortic stenosis (AS) is a progressive disease, with no pharmacological treatment. The prevalence of diabetes mellitus (DM) among AS patients is higher than in the general population. DM significantly increases the risk of AS development and progression from mild to severe. The interplay between AS and DM's mechanism is not entirely known yet.

**Main Body:**

The increased accumulation of advanced glycation end products (AGEs) was linked to increased valvular oxidative stress, inflammation, expression of coagulation factors, and signs of calcification, according to an analysis of aortic stenotic valves. It is interesting to note that in diabetic AS patients, valvular inflammation did not correlate with serum glucose levels but rather only with long-term glycemic management markers like glycated haemoglobin and fructosamine. Transcatheter aortic valve replacement, which has been shown to be safer than surgical aortic valve replacement, is advantageous for AS patients who also have concurrent diabetes. Additionally, novel anti-diabetic medications have been proposed to lower the risk of AS development in DM patients, including sodium-glucose cotransporter-2 inhibitors and glucagon-like peptide-1 receptor agonist that target reduction of AGEs-mediated oxidative stress.

**Conclusions:**

There are little data on the effects of hyperglycemia on valvular calcification, but understanding the interactions between them is essential to develop a successful treatment strategy to stop or at least slow the progression of AS in DM patients. There is a link among AS and DM and that DM negatively impacts the quality of life and longevity of AS patients. The sole successful treatment, despite ongoing efforts to find new therapeutic modalities, involves aortic valve replacement. More research is required to find methods that can slow the advancement of these conditions, enhancing the prognosis and course of people with AS and DM.

**Graphical Abstract:**

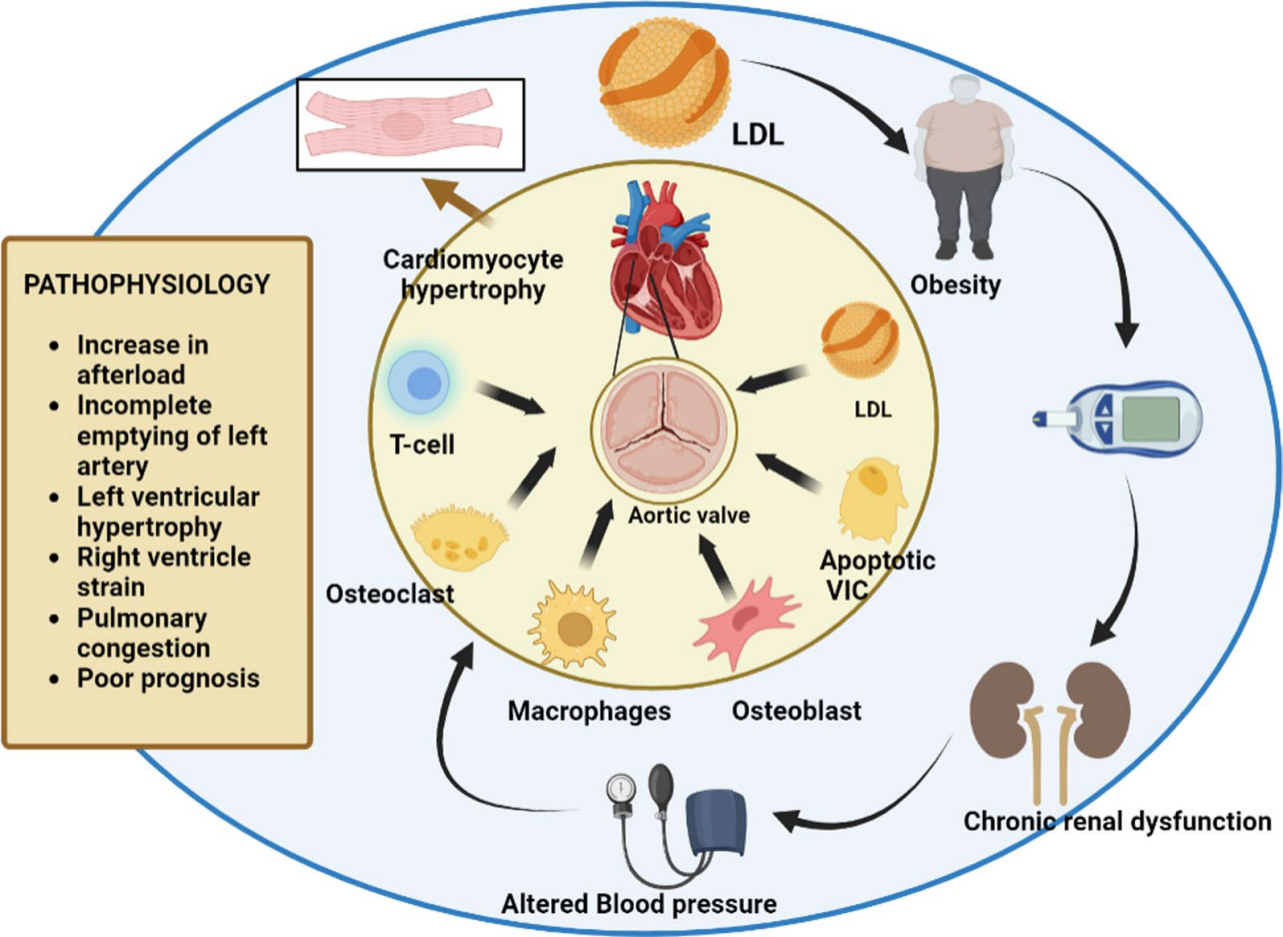

## Background

Globally, valvular heart disorders are a significant public health burden. Aortic valve stenosis is currently the most prevalent valvular condition in Western nations as rheumatic disease prevalence has declined. Its prevalence rises with age, affecting 0.2% of people between the ages of 55 and 64 [1] and 2–7% of those over the age of 65 [2]. By the year 2030, there will likely be 4.5 million instances of AS worldwide [3]. The most prevalent valve disease, calcific aortic stenosis (AS), causes the valves to become thicker and stiffer, and in certain cases, nodular deposits develop, reducing valve function. This may lead to concurrent stenosis and valve regurgitation.

Calcific AS is a chronic condition that worsens with ageing [4, 5]. It affects 0.2% of adults between the ages of 50 and 59 and 9.8% of those between the ages of 80 and 89. The frequency of calcific AS has grown as the vast majority of the population has aged, sparking several advancements in its care. Novel prosthetic valves have also been developed as a successful therapy for calcific AS in addition to improved diagnostic imaging methods. Pharmacotherapy has not yet been demonstrated to stop the illness from progressing or to stop overall calcification process [6, 7].

The advancement of both atherosclerosis and DAS is thought to be influenced by a number of illnesses and conditions, including hypertension, hypercholesterolemia, and diabetic mellitus (DM). The development of atherosclerosis is accelerated by both type 1 and type 2 diabetes (T1DM and T2DM), which is caused by both the hyperglycemia that is created as well as the related insulin resistance, dyslipidaemia, etc. In addition, T2DM significantly increases lipid build up and the inflammatory response. These pathways, which are linked to hypertrophic left ventricular remodelling, elevated left ventricle mass, increased left ventricle end-systolic dimension, and decreased systolic function, also influence the development of DAS. All of these processes show that DM negatively affects cardiac function, and it has been seen that AS patients having DM diabetes have considerably poorer left ventricular diastolic performance [8]. As a result, DM adversely affects the diastolic and systolic functioning of the myocardium directly, and it also indirectly impacts cardiac function by causing comorbidities such coronary artery disease. These processes are what make DM more likely to cause Heart Failure (HF) in people with AS.

### Aortic valve structure and calcification

Although aortic stenosis (AS) and aortic valvular sclerosis (AVS) were formerly regarded to be two distinct conditions, they are now understood to represent different phases of the same disease. The first symptom of this condition is a thickening of the valve brought on by lipocalcified deposits, which progresses to a decrease in the valve opening and eventually results in hemodynamically significant stenosis.

### Anatomy

**Fig. 1:**
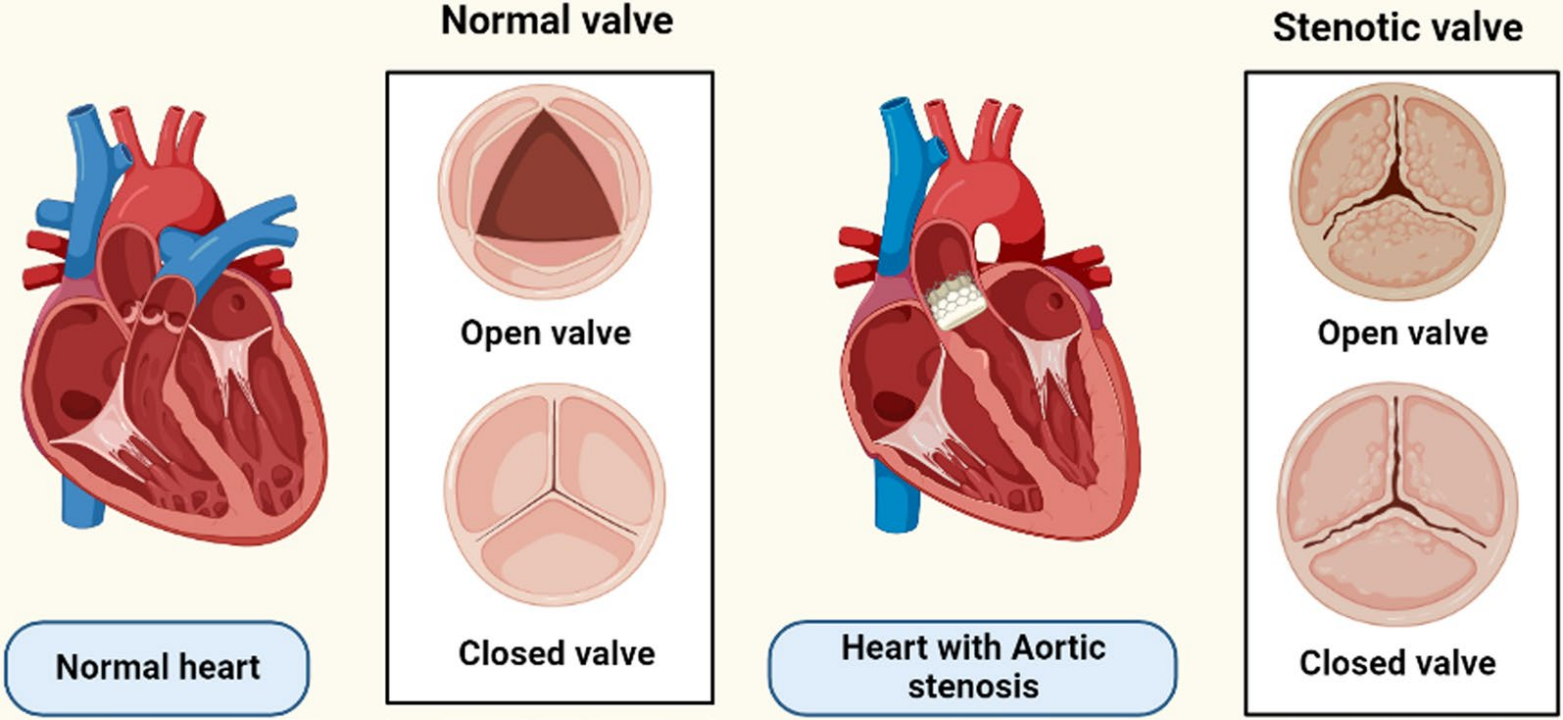
Diagrammatic representation of normal aortic valve versus aortic Stenosis

**Fig. 2:**
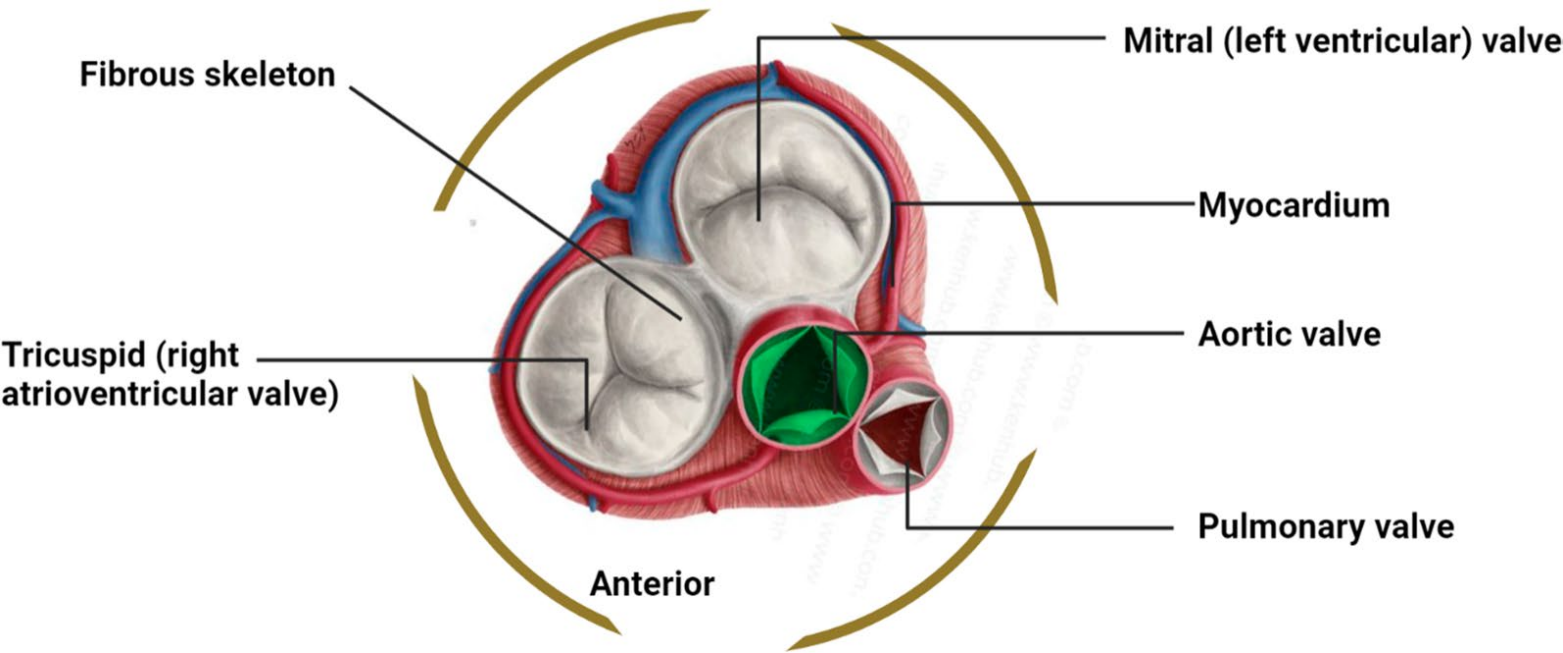
Anatomy of Aortic valve

Of the four heart valves, the aortic valve is one (mitral, pulmonary and tricuspid the other three). It is located in the aortic root, which connects the left ventricular outflow tract (LVOT) also with ascending aorta, as well as its primary job is to allow blood to flow out from left ventricle (LV) to the systemic circulation when the valve opens and to stop blood regurgitation in the LV when the valve shuts during diastole [9]. Figure illustrates how the aortic valve, which is connected to all of the cardiac chambers together with the other three valves, is in the heart's centre as seen from above [10]. The heart's fibrous skeleton, a structure consisting of thick fibrous tissue which encircles all four valves, stabilises and supports the heart valves. The three aortic leaflets or cusps, the three sinuses of Valsalva, and the triple fibrous interleaflet triangles make up the aortic valve. The luminal surface of a wall of the aortic root creates three bulges, each of which corresponds to a different one of the three sinuses of Valsalva [10]. The sinotubular junction, also known as the aortic root-to-ascending aorta transition point, is formed by the ridge on top of the sinuses and is a circular, well-defined ring consisting of thicker aortic wall [11]. The sinuses offer the space required for the leaflets to open completely during systole [12]. Both left and right coronary arteries' orifices are also often located in two of the sinuses, while it is not uncommon for them to be located above the sinotubular junction. In any event, the coronary artery's opening that is situated within or close to each sinus gave the sinuses of Valsalva their names (right, left, and noncoronary) [13] (Figs. 1, 2).

#### Cardiovascular/aortic valve calcification

Cardiovascular calcification, a disordered mineral metabolism condition, is not a brand-new problem. In fact, according to some studies, it has existed ever since Ice Age [14]. Atherosclerosis & cardiovascular calcification are accelerated by hyperlipidemia, metabolic disorders, end-stage renal disease, diabetes mellitus, and advancing age. The aorta, coronary arteries, peripheral arteries, & aortic valves are the principal areas affected by ectopic mineralisation, with fully formed bone being seen in atherosclerotic plaques and stenotic aortic valves [15]. Cardiovascular calcification, once thought that it is passive degenerative illness, is now understood to be an active process that may follow a similar pathway to bone creation. However, because age and lifestyles are still important variables, the burden of this condition is expanding along with the average population age, which has a significant financial impact on society.

#### Arterial calcification

Cardiovascular calcification acts as a biomarker for atherosclerotic coronary artery diseases and is linked to an increase in cardiovascular events. It is commonly assessed and measured in patients utilising imaging modalities like computed tomography (CT) [16]. It has been demonstrated that coronary artery calcification grading generated by CT can accurately predict future coronary heart disease occurrences [17]. Acute thrombosis and potentially fatal myocardial infarction can result from microfractures that are brought on by arterial microcalcifications that are present in the thin (≤ 65 µm) fibrous cap that covers the necrotic centre of atherosclerotic plaques [18, 19]. However, calcification remains generally neglected condition, and there are currently no effective anti-calcification medicines, despite data suggesting that microcalcifications in fibrous tissue caps raise the risk of plaque rupture.

#### Aortic valve calcification

Numerous pieces of evidence point to the similarities between arterial and valvular calcification. Lesions comparable to those present in atherosclerotic plaques have been seen in clinicopathological examinations of human stenotic aortic valves [20], whereas atherosclerotic-like lesions have been found inside the aortic valve leaflets of rabbit & mouse animal models of atherosclerosis [21, 22]. Further supporting the correlation between aortic valve stenosis & coronary atherosclerosis is their epidemiologic risk factors [23]. Aortic valve stenosis, a most frequent kind of heart valve illness, or significant calcification with decreased leaflet mobility is both possible symptoms of calcific aortic valve disease [24]. As a result, calcification is a reliable indicator of disease progression in individuals with aortic stenosis who were previously asymptomatic. As a result of aortic valve stenosis, over 85,000 people in the USA and 275,000 patients globally yearly have valve replacement surgery. This invasive and expensive procedure is the only viable remedy [8].

### Molecular link/ ominous combination with diabetes mellitus

**Fig. 3:**
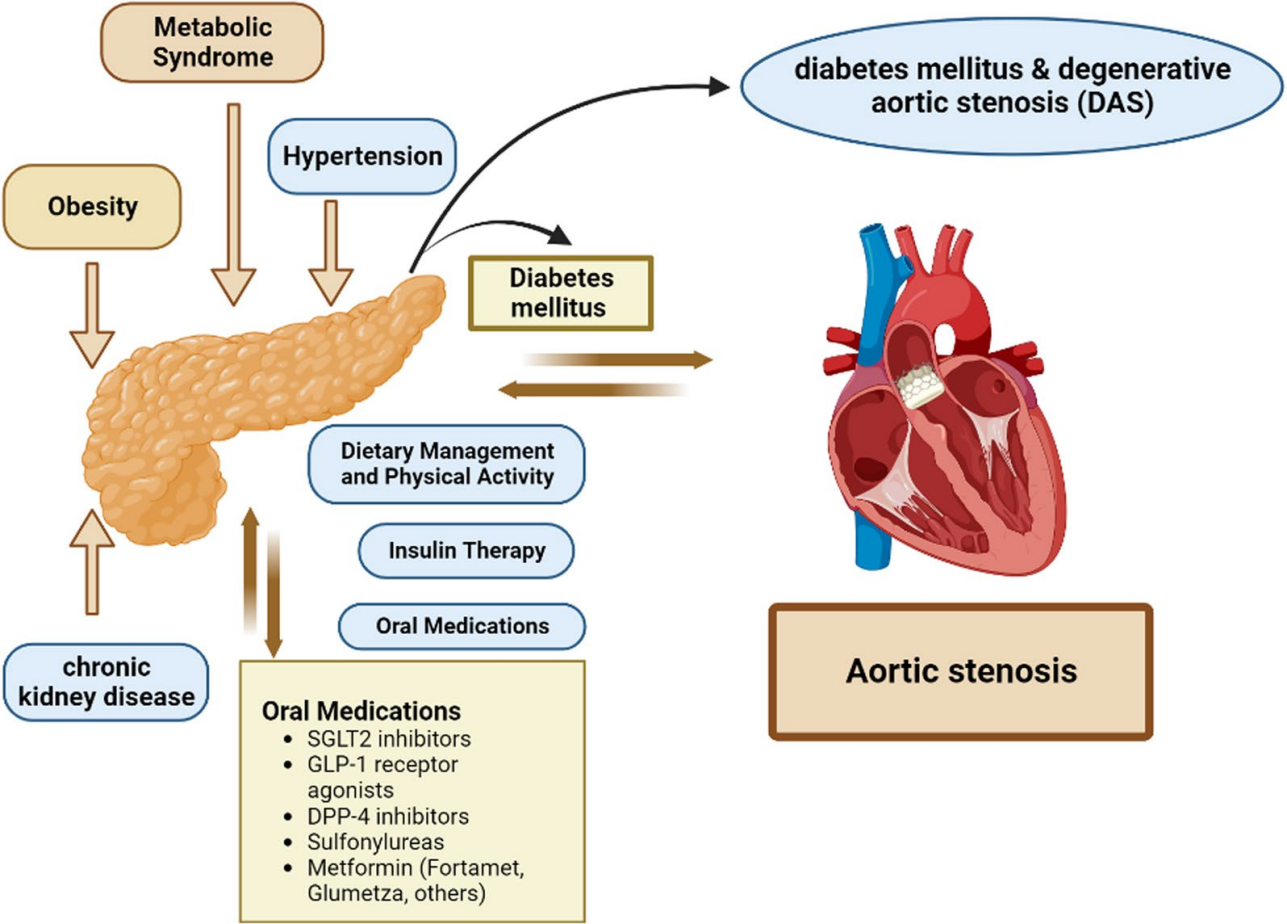
Correlation between Diabetes Mellitus and Aortic Stenosis

Despite the established link between DM and atherosclerosis and the parallels among AS and atherosclerosis, there is little information on how hyperglycemia affects calcification and inflammation of the valves. However, it has been suggested that hyperglycemia, along with other metabolic variables, may start or at least exacerbate valvular calcification through some kind of complicated process involving interactions between vascular and inflammatory cells [25]. According to immunohistochemistry examination of AS valves, concurrent DM was linked to an elevated proportion of C-reactive protein-positive regions and was correlated with the proportion of TF-positive areas [26]. Also indicated as a factor in the accelerated course of AS is an increase in valvular protein glycation brought on by an aggregation of enhanced glycation end products (AGEs) [27, 28]. A heterogeneous set of lipids or proteins called AGEs have had their free amino groups attached by reducing sugars, causing irreversible glycation. Through the cross-linking of intracellular and extracellular matrix proteins and binding to the AGE receptor on the cell surface (RAGE), which has an impact on a variety of cellular functions, AGEs alter tissue structure and function. AGEs are formed more quickly when exposed to higher blood glucose levels in DM [29, 30]. The build-up of AGEs in aortic valves led to the osteoblastic development of VICs, as demonstrated by the rabbit & mouse models of AS [31]. In addition, the rabbit model of AS exhibits increased oxidative stress & NF-Kb overexpression due to higher AGE concentrations [32]. Additionally, it has been suggested that the production of inflammatory cytokines including TF by monocytes and macrophages is mediated by RAGE-induced NF-B activation. A 6.6- and 12-fold rise in valvular & plasma AGEs was linked with AS severity, defined by the decreased aortic valve area, in AS patients with concurrent DM. This impact of AGEs on AS development was recently demonstrated in these individuals [32]. Comparatively to non-diabetics, diabetic AS patients showed RAGE expression that was 1.8 times greater in aortic stenotic valves and 1.3 times higher in plasma. Notably, only plasma RAGE levels were associated with the severity of AS, whereas the impact of hyperglycemia on AS severity was minimal in individuals with well-controlled type 2 DM (HbA1c 7%) [9]. In addition, diabetic AS patients exhibited higher NF-B valvular expression along with increased valve expression of coagulation factors II and Xa and BMP-2, a calcification marker, as compared to non-diabetic AS patients [33]. This finding was supported by an in vitro investigation employing VICs obtained isolated stenotic aortic valves, wherein calcification was avoided by inhibiting either reactive oxygen species and NF-B [34]. It is interesting to note that in diabetic AS patients, valvular NF-B expression was connected with both the severity of the AS and the long-term glycemic management indices HbA1c and fructosamine. Furthermore, TF and FVIIa-antithrombin complex plasma concentrations were noticeably greater in AS patients with poorly managed type 2 DM classified as HbA1c ≥ 6.5% (Fig. 3).

### Risk factors associated with DM for aortic stenosis

**Fig. 4:**
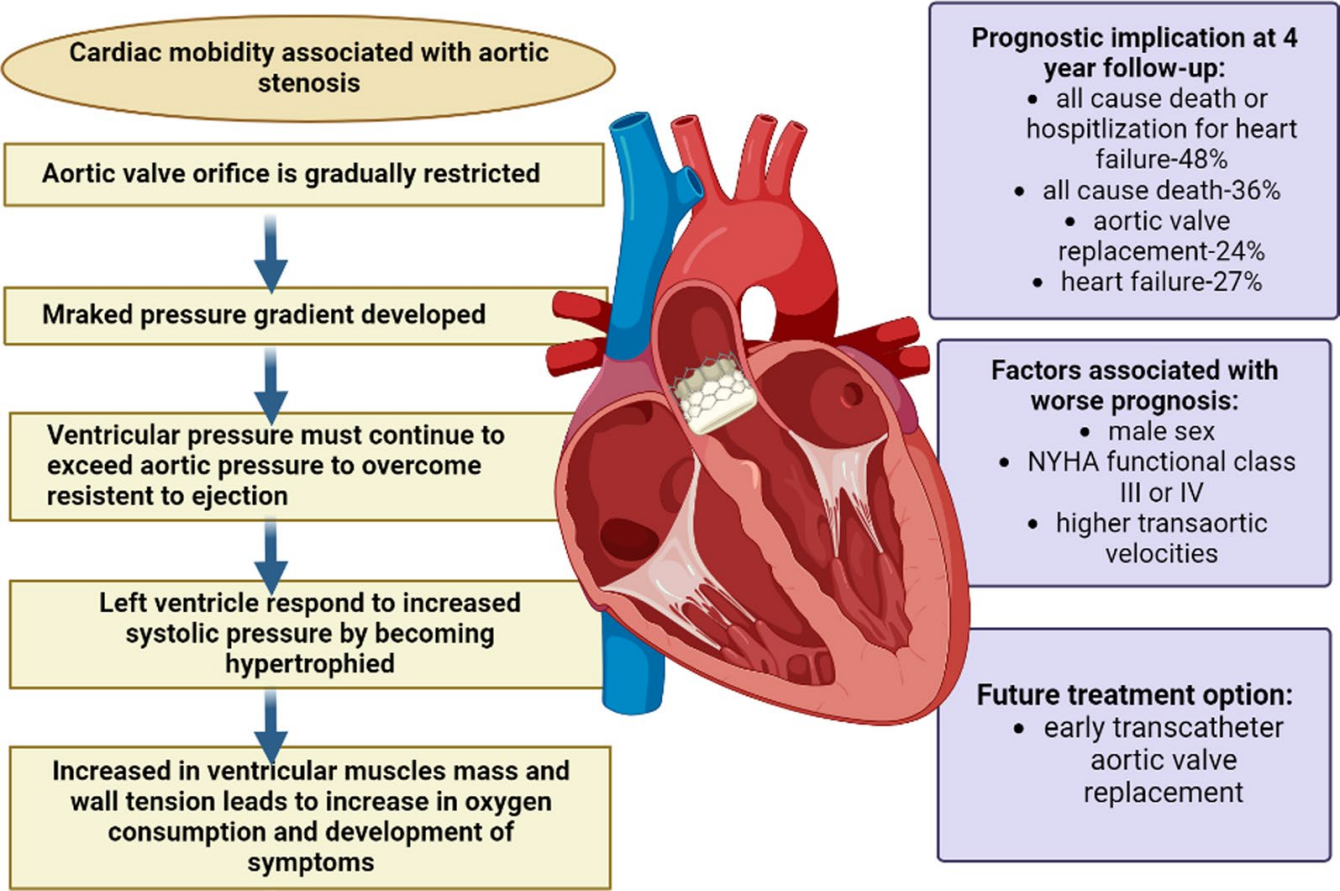
Cardiac morbidity associated with aortic Stenosis

Although it is now apparent that DAS and DM are linked, several studies have shown quite diverse rates of diabetes in the DAS population. DAS was recorded in 41% of diabetics, despite another research placing this figure closer to 30%. In fact, only 5% of DM patients had AS according to the SALTIRE research, and there is little agreement on the proportional frequency of diabetes among both the overall population as well as in DAS patients. Diabetes was present in 20% of individuals with severe AS in 2003, compared to 18% of the age-related controls [35]. Although individuals with AS were significantly older and had higher rates of arterial hypertension, obesity, diabetes, hypercholesterolemia, and cardiovascular disease, there were differences in the prevalence of diabetes while comparing individuals with or without changes in AV structure (3.8 and 1.3%, respectively). On the other hand, research including bigger cohorts shows that people with AS have a much greater prevalence of diabetes. DM was found to increase the risk of developing AS in the Multi-Ethnic Study of Atherosclerosis (MESA), which included 5723 individuals (OR 2.06; 95% CI 1.39–3.06). [36]. In addition, the Cardiovascular Health in Ambulatory Care Research Team (CANHEART) found that having DM increased the chance of developing AS (HR—hazard ratio 1.49; 95% CI 1.44–1.54) after conducting a population-based observational analysis on a cohort of 9.8 million persons. Studies have also looked at how diabetes affects the quality of life, calcification development, and survival of AS patients. Although this is still debatable, the majority of them suggest that DM affects AS patients’ event-free survival (EFS), regardless of whether they received conservative care or percutaneous or surgical intervention. For instance, in the MESA trial, persons who had AS at baseline were not related with DM, whereas in the Helsinki Aging Study, DM was not a standalone prediction of AV calcification. On the other hand, in patients suffering from severe AS, DM was discovered to be a significant predictor of poor result after interventions and an independent driver of cardiovascular death [37]. Diabetes has also recently been discovered to be a standalone predictor of AS-related outcomes [38]. This intriguing study developed the CURRENTAS risk score, which included DM, haemodialysis, any concurrent valve disease, left ventricle ejection fraction (LVEF) ≥ 60%, haemoglobin 11 g/dL, and chronic lung disease as independent predictors of AS-related occurrences 1 year since diagnosis in asymptomatic patients with severe AS (Fig. 4).

The current debates may be a result of the varied baseline clinical features and nationalities of the groups analysed in each research. The incidence of DM is affected by various cultures and lifestyles; hence nationalities are crucial. In actuality, a higher body mass index (BMI), a risky diet, & ambient particulate matter contamination are the three leading risk factors for diabetes [39]. The prevalence of diabetes worldwide grew between 211.2 million in 1990 to 476.0 million in 2017, a rise of 129.7%, when analysing the data from this research. [40].

### Pathogenesis of CAVD

The aortic valve side experiences a focal stiffening of the valves in the early stages of the illness, which starts just at subendothelial level and thereafter progresses to the outermost or fibrous layer. Long-lasting flexibility ensures that the opening mechanism of these valves remains unaffected. The aortic valve becomes substantially stiffer and has a significantly smaller valvular area as a result of the regions of thickening converging into huge calcified masses over time. This interferes with the valve's normal function. Patients with bicuspid aortic valves, the most common congenital aortic valve defect, as well as those with tricuspid valves are both affected by sclerosis and valvular stenosis. It is challenging to determine the prevalence of bicuspid aortic valves; however, it is thought to afflict 1 to 2% of the general population. Aortic valve replacement is necessary one to two decades earlier in individuals having bicuspid aortic valves than in those with trivalve aortic valves because up to 70% of these people have valvular stenosis. It is believed that in these cases, traumatic cusp degeneration culminates in fibrous degeneration and subsequently leads to valve calcification [41].

#### Mechanical stress

The whole cardiac cycle places a significant mechanical strain on the aortic valve. As a result of this mechanical strain, valves constantly regenerate, which encourages valve disease. The leaflet's zones of flexing experience the most stress. The process starts in these regions of higher mechanical stress, while bending force, pressure, and shear forces will cause damage to the structural integrity of the leaflet tissue and induce calcification. At this time, it is believed that mechanical stimulation contributes significantly to valvular calcification.

#### Endothelial dysfunction

This mechanical load on the valve’s flexion zone leads to endothelial dysfunction by eroding the endothelium. Long believed to be a layer of cells that only served coating duties, valve endothelial cells. The loss of such capabilities is a crucial factor in the development of atherosclerosis. Today, that layer is thought of as a barrier that guards against metabolic, mechanical, & inflammatory assaults [42]. Enhanced cell permeability, adhesion, & proliferation are encouraged by endothelial injury, which makes it easier for lipids to diffuse into the interstitial valvular tissue and deposit there if there is inflammation and calcification.

#### Lipoprotein deposit and oxidative stress

In the chain of cellular signalling that results in valvular calcification, lipid deposit is a key initiator. Low-density lipoproteins (LDLs) & lipoprotein A are two of the lipoproteins implicated in the process. These are molecules involved in atherosclerosis that oxidise and generate free radicals, which are extremely cytotoxic and can also activate inflammation and mineralisation [43].The reduction of normal endothelium-level nitric oxide levels [44] and the noticeably elevated levels of free radicals like superoxide and oxygen peroxide [45], which are explained by a change in nitric oxide synthetase's typical function, respectively, are indicators of the increased oxidative stress during this process.

#### Inflammation

T lymphocytes and macrophages are the primary inflammatory cells under the microscope in CAVD [46]. These cells invade and accumulate in the sub-endothelium, which increases the production of cytokines that are pro-inflammatory and other enzymes that break down the extracellular matrix [47]. They can also cause fibroblasts to change into myofibroblasts with an osteoblastic phenotype, which promotes the development of calcium and bone nodules [47]. This may be shown by the significant rise in the cytokine’s interleukin-1 (IL-1) and tumour necrosis factor alpha (TNF-α) seen in calcified AS [48].

Furthermore, it should be noted that CAVD has pathological angiogenesis, which is encouraged by inflammatory mediators because they enhance growth factors as well as endothelial transformation, which can lead to fibrosis and the advancement of calcification [49].

### Alteration of the extracellular matrix and calcification

The extracellular matrix is remodelled and calcification occurs in the latter stages of the illness. The release of inflammatory cytokines causes an increase in cellular proliferation, which is expressed as increased matrix synthesis, and activates the extracellular matrix metalloproteinases, that also favour the breakdown of all of the matrix's components as well as directly promote the propagation of fibroblasts, which increases fibrosis [50]. Calcium build-up and increased fibrosis both contribute to the thickness and stiffness of the valves, which causes valvular stenosis. The complicated active process of aortic calcification involves the synthesis of proteins that encourage tissue calcification. In actuality, extracellular matrix proteins including osteocalcin, osteopontin, and osteonectin—which are typically found in bone—can also be present in calcified valves.

### Activation of the renin–angiotensin–aldosterone system

Renin–angiotensin–aldosterone system alterations in CAVD have a role in the pathophysiology of the lesion. Angiotensin-converting enzyme (ACE), angiotensin II, and angiotensin I receptors are all affected by these changes, which are linked to an increase in LDL absorption, inflammation, and a profibrotic state [51]. It has long been believed that treating these individuals with medications that inhibit the renin–angiotensin–aldosterone cascade is advantageous, and a retrospective analysis did indeed find that the course of calcification was slowed [52]. Unfortunately, there is currently no evidence that using this class of medications can improve these patients' prognoses or slow the hemodynamic course of their illness [53].

### Genetic factors

There is currently proof that genetic factors influence the progression of CAVD. Patients with the condition have been shown to have a number of genetic variations, including mutations in the genes encoding for the vitamin D receptor [54] and the apolipoproteins that determine an individual's lipid load [55]. The polymorphism of the transcriptional factor NOTCH 1, which controls the process of osteogenic differentiation, is another polymorphism that has received substantial research. Osteoblast differentiation is normally inhibited by this system, and therefore alterations at this level favour osteoblast differentiation, favouring calcification as well as the development of CAVD.

### Molecular pathogenic pathway of aortic stenosis

**Fig. 5:**
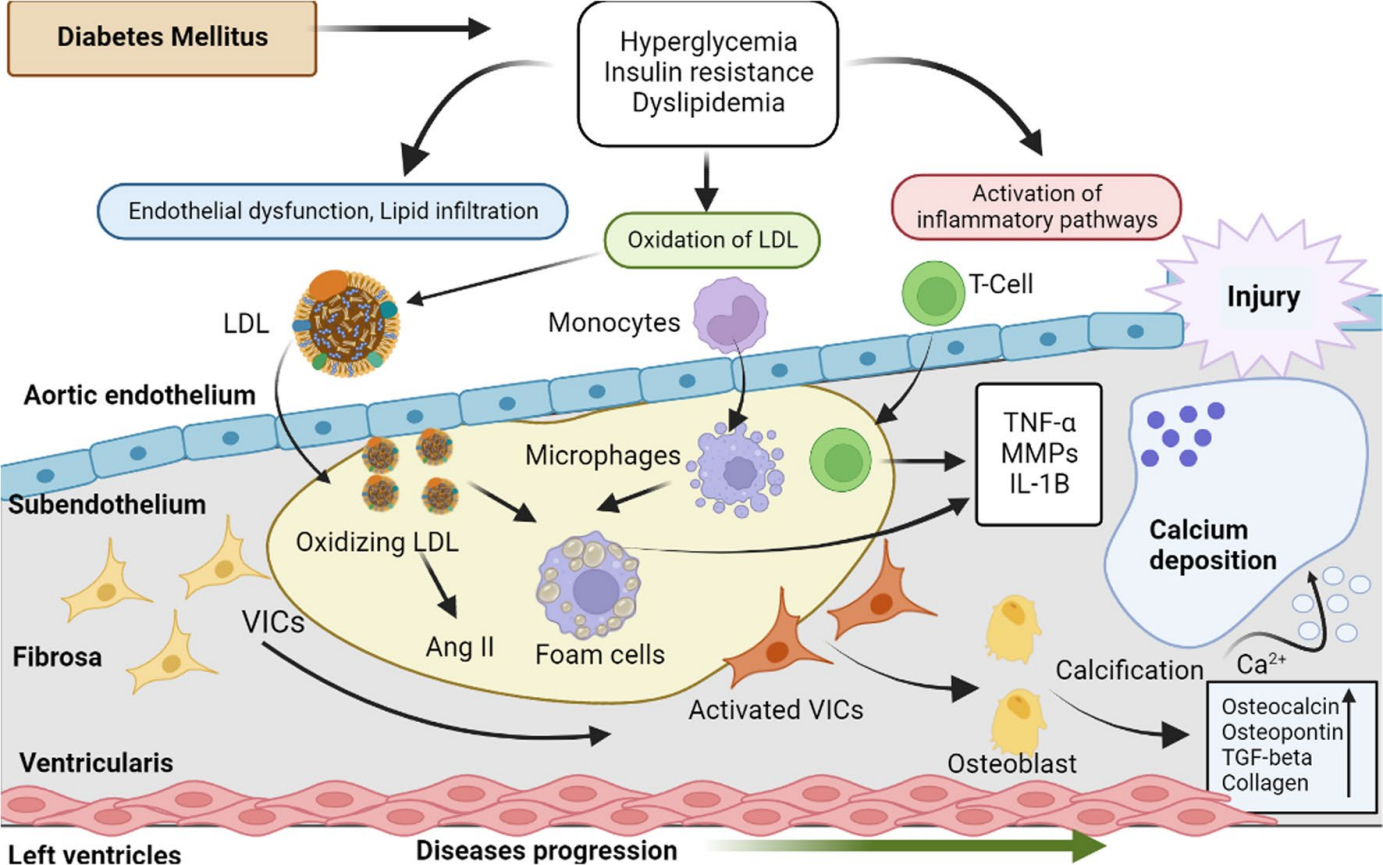
Diagrammatic representation of the pathophysiological connection of diabetes mellitus and aortic stenosis

AS, originally known as aortic valve sclerosis, is characterised by high shear stress-induced damage to the valve endothelium [56] as well as subendothelial lipid and lipoprotein build-up and increased oxidative stress [57]. These events cause the valve leaflets to become calcified by driving cell-dependent systems that control the calcium load on them and activating the local inflammatory response [58]. Valvular interstitial cells (VICs), a cell type that predominates in aortic valves and is responsible for the pathobiology distinctions between atherosclerosis and AS, play a significant role in valvular calcification under both pathological settings [59]. VICs undergo epigenetic changes to develop to osteoblast-like cells, at least partially [60]. According to one definition, valvular calcification results from closely controlled mechanisms that result in the ordered deposition of osteoblast-like cells in extracellular matrix [60]. These activated cells respond to common osteogenic mediators such bone morphogenetic proteins and members of the transforming growth factor *β* superfamily (BMPs) [61, 62] (Fig. 5).

Numerous pathogenic processes, including inflammation, low-density lipoprotein (LDL) oxidation, and endothelial dysfunction, are sparked by hyperglycemia, insulin resistance, and dyslipidemia. This causes the extracellular matrix to restructure and stimulates valve interstitial cells (VICs). Calcium crystals are produced by cells that resemble osteoblasts, and they ultimately lead to macrocalcification. Angiotensin II (AngII), tumour necrosis factor (TNF), matrix metalloproteinases (MMPs), interleukin (IL), and transforming growth factor (TGF) are all abbreviations.

Aortic calcification is a multi-stage condition that may be broken down into two separate phases: an early initiation phase and a later propagation phase, each of which is defined by a unique mechanism. The initial stage, known as aortic sclerosis, resembles atherosclerosis, and both disorders have comparable risk factors (age, male gender, smoking, hypertension, dyslipidemia, metabolic syndrome) [63]. Endothelial damage on the aortic side of the valve, caused by higher mechanical stress and decreased shear stress, is symbolic of the starting event. The infiltration of monocytes, mast cells, T cells, and lipoproteins (such low-density lipoprotein (LDL), lipoprotein(a)), which promotes inflammation and fat build-up, is made easier by the loss of endothelial integrity [64]. Monocytes become activated to become macrophages once they are in the sub endothelium, T cells express pro-inflammatory cytokines (interleukin (IL)-1, IL-6, and tumour necrosis factor (TNF)-), and LDL is oxidised to become oxidised LDL (oxLDL), which is recognised by macrophage scavenger receptors and gives rise to foam cells. Additional oxidative stress and an inflammatory response are brought on by these mechanisms. Although lipid deposition and inflammation may play a significant role in the development of the illness, they play a less significant function during the propagation phase, which would be primarily marked by fibrosis & calcification.

The most common group of cells in the aortic valve (AV) are valvular interstitial cells (VICs), which are dispersed all across the three extracellular matrix (ECM) layers and contribute to the development of CAVS [65]. Valvular endothelial cells (VECs), which further appear to be important in disease progression, form a monolayer around the tissue [66]. Although VICs are normally dormant, transforming growth factor (TGF) and pro-inflammatory cytokines have the ability to activate them and turn them into myofibroblast-like cells [67].

The ECM components, particularly collagen fibres, are produced and deposited in greater quantities when VICs are activated. The excessive build-up of disordered collagen fibres causes the tissue to undergo fibrotic remodelling, which makes the leaflets stiffer. VICs may also undergo apoptosis during this phase, releasing apoptotic bodies which serve as microcalcification nucleation sites [68]. Valvular calcification, which is facilitated by dystrophic calcification and biomineralisation, is another significant step of the propagation phase. The first mechanism involves the passive accumulation of amorphous hydroxyapatite (HA) crystals on apoptotic bodies and in the damaged ECM. These crystals are made of calcium and phosphate ions. The second step, which resembles the development of skeletal bone, is fueled mostly by osteogenic differentiation of VICs, which is encouraged by a number of signalling pathways, particularly RANK/RANKL [69], ENPP1 [70], &Wnt/-catenin [71]. The osteoblast-like phenotype can release phosphate- & calcium-rich matrix vesicles, which eventually aggregate and serve as scafolds for the deposition of HA crystals [72, 73]. It is characterised by increased expression of osteogenic markers, including RUNX2, BMPs, osteocalcin, osteopontin, and bone sialoprotein. These vesicles also include ectonucleotidases, which produce inorganic phosphate ions through endogenous sources and aid in the growth of HA crystals. Massive deposits of minerals resembling bone are formed in the valvular ECM as a result of simultaneous dystrophic calcification and biomineralisation.

### Comorbidities among diabetes mellitus & degenerative aortic stenosis (DAS)

Multimorbidity, the presence of multiple or more pathological illnesses, is a top concern for world health. Multimorbidity is more common than previously thought, with estimates ranging from 55 to 98 percent, and it is linked to worse outcomes for illness management and treatment choices [74]. As a result, there is a need to enhance these patients' follow-up, which is a significant public health concern. The QoL of these individuals will be improved, and the use and expense of healthcare services will be decreased by identifying similar patterns of multimorbidity [75]. These comorbidities, which are frequent in DM patients and include chronic kidney disease (CKD) as well as coronary artery disease, have an independent impact on the patient's life expectancy irrespective of the valve disease, which makes them relevant in risk–benefit evaluations. Therefore, prevalent comorbidities may make DM's impact on AS patients worse. Importantly, a significant portion of individuals who had severe DAS or T2DM had one or more comorbidities, including hypertension, dyslipidemia, or obesity [76], some of which are frequent in people with DAS and T2DM [77]. This puts individuals who have both DAS and T2DM at an increased risk of experiencing a negative event (Table 1).

**Table 1:** Case study data

Study Title	Conditions	Interventions	Locations
The Medtronic Transcatheter Aortic Valve Implant System (CoreValve System Family) Post Marketing Surveillance (PMS) (CoreValve India PMS)	Symptomatic Aortic Stenosis	Device: CoreValve System Family	Eternal Heart Care Centre Jaipur, Rajasthan, India
Portico India Clinical Trial	Aortic Valve Stenosis Aortic Valve Failure Aortic Insufficiency Aortic Stenosis	Device: Transcatheter Aortic Valve Replacement	Apollo Hospital Chennai, India Rajasthan Hospital Jaipur, India Seth GS Medical College & KEM Hospital Mumbai, India Christian Medical College & Hospital Vellore, India
Prospective Randomized On-X Versus SJM Evaluation Trial (PROSE)	Heart Valve Disease	Device: On-X Heart valve replacement Device: SJM Heart valve replacement	Kaiser Foundation Hospital Honolulu, Hawaii, United States
PRESERVE-MITRAL Post-Market Registry	Mitral Valve Disease	Device: Profile 3D™ and CG Future® annuloplasty system	SAL Hospital & Medical Institute Ahmedabad, Gujarat, India
Effect of Ivabradine on Heart Rate & Effort Tolerance in Mitral Stenosis in Sinus Rhythm	Mitral Stenosis	Drug: Ivabradine Drug: Atenolol	G. B. Pant Hospital New Delhi, Delhi, India

*Sources: clinicaltrials.gov*

#### Hypertension

Pathophysiological factors that contribute to hypertension include the renin–angiotensin–aldosterone system (RAAS) being upregulated, endothelial dysfunction/oxidative stress, sympathetic nervous system (SNS) activation, and immune system activation [78]. Patients with DM frequently have hypertension, which has been linked to faster DAS development. [79]. While T2DM patients' hypertension is linked to insulin resistance and atherosclerosis, T1DM patients' is linked to diabetic nephropathy and diabetic nephropathy. Adverse cardiovascular events, including macrovascular and microvascular complications like myocardial infarction, coronary artery disease, stroke international, or nephropathy, are brought on by hypertension in both diabetes people and DAS [80]. Furthermore, compared to normotensive patients with DAS, those with hypertension had a 56% greater incidence of ischemic cardiovascular events and a higher death rate, according to the SEAS (Simvastatin Ezetimibe in Aortic Stenosis) trial [81]. Despite the lack of research on particular antihypertensive medications for DAS patients, it is essential to control and treat their hypertension properly in order to lower their risk of cardiovascular disease and avoid cardiovascular consequences [82].

#### Chronic kidney disease

9.1% of the world's population has chronic kidney disease (CKD), and patients with CKD have a high incidence of DAS, which causes calcification to advance more quickly than in people with normal renal function. Additionally, having CKD increases “all cause” and cardiovascular mortality in DAS patients, and these patients have a greater perioperative mortality following AV replacement. [83].

Regardless of the stage of CKD, AV replacement is likewise strongly linked to a decrease in mortality after 5 years. As an alternative, one of the most prevalent consequences of diabetes affects 20–40% of diabetic people. Additionally, diabetic nephropathy raises the risk of cardiovascular disease including death. To lower these consequences & mortality in diabetic patients, glycaemic control and effective treatment of cardiovascular risk factors are required: dyslipidemia and atherosclerosis. It is currently reported that more than 50% of adults globally have dyslipidemia, which is widely known as a cardiovascular risk factor. [84].

Dyslipidemia shares pathogenic processes with the early stages of DAS and leads to lipid build-up inside the arteries that accelerates atherosclerosis. Several studies have also shown a connection between calcium deposition and DAS risk as well as lipoprotein levels [85]. Contrarily, dyslipidemia and diabetes frequently co-occur, and in DM patients, hyperglycemia and insulin resistance speed up atheroma development. Additionally, insulin resistance influences not only the development of atherosclerosis but also the development of hypertension and dyslipidemia, raising the risk of AS. Despite the fact that scientific models predict statin-based lipid-lowering treatment may slow the course of AS, these advantages were not shown in three retrospective clinical investigations [86].

#### Obesity

Another factor that may raise the risk of AS is obesity, which is a complex condition. Although some research [87] yielded contradictory findings, a link between BMI or waist circumference and the likelihood of having DAS has been suggested. Furthermore, a recent study found a correlation between the risk of DAS & replacement surgery and human hereditary obesity [88]. Additionally, a high BMI increases the chance of developing diabetes, and even more than 90% of DM patients are overweight or obese. Obesity makes DM problems worse when it is accompanied by insulin resistance [89]. Obesity and DM together worsen the risk of AS since, as previously mentioned, DM alone causes to rapid progression of DAS.

#### Metabolic syndrome

The development of several cardiovascular disease and type 2 diabetes risk factors is known as the metabolic syndrome. Obesity, DM, and the metabolic syndrome are all linked to cardiovascular problems. Insulin resistance and abdominal obesity are the main causes of metabolic syndrome [90]. The simultaneous occurrence of these changes raises the probability of unfavourable outcomes and makes metabolic syndrome associated with the advancement of DAS regardless of conventional risk factors. Additionally, research has shown that early calcification and mechanical and anatomical alterations to the AV are also associated with metabolic syndrome [91]. On the other hand, other investigations claim that people with metabolic syndrome have a higher chance of acquiring diabetes [92]. Decreases in metabolic syndrome status are therefore required to lower the risk of DM and, consequently, the risk of DAS. Finally, increased DAS and DM comorbidities raise the likelihood of complications, which has an impact on patient care. So, in order to reduce the incidence of adverse events and design effective treatment plans, a multifaceted approach is required.

### Imaging technique involved in aortic stenosis

Techniques for cardiac imaging are crucial in the investigation of CAVD. They are quite useful for prognosis in addition to establishing the diagnosis and determining the severity of the condition. This enables planning for the ideal time for valve replacement by allowing evaluation of the potential functional consequences and follow-up of patients at risk. The cornerstone of these imaging methods is still echocardiography. To overcome the technical limitations of echocardiography and provide additional information on certain anatomical and functional aspects that are particularly important when thinking about transcatheter aortic valve implantation (TAVI) as well as surgical repair techniques, other recent technologies, including such cardiac magnetic resonance imaging as well as computed tomography, have also shown their utility [93].

#### Echocardiography

In order to examine CAVD, transthoracic echocardiography (TTE) is the preferred technique. It is a non-invasive, secure, and frequently used technology that enables extremely early identification of valvular abnormalities brought on by calcium deposition, which are characterised by valve thickness or sclerosis. The illness does not initially significantly affect hemodynamic, but as it advances, there is a large increase in valve stenosis with serious functional consequences, which typically occurs at the same time as the onset of symptoms.

TTE gives us additional information about other crucial parameters, such as ventricular function, cavity size, and perhaps pressure at the level of the pulmonary artery, which may change in response to pressure overload and as a result of a complicated process of ventricular remodelling [43]. TTE not only enables us to assess valve morphology, the cause of the stenosis, and the severity of the condition [94]. Given everything mentioned above, current clinical practise recommendations recommend scheduling routine echocardiographic follow-up for these patients, and this is a key consideration when deciding whether to replace the aortic valve [95].

#### Cardiac magnetic resonance imaging (CMRI)

With no X-ray exposure, cardioresonance is an emergency, non-invasive, safe technology that is also highly accurate and spatially resolution. It is now the reference approach for the non-invasive evaluation of LV dimensions and mass as well as both global and regional left ventricular performance due to its high accuracy and consistency, even in patients with subpar echocardiographic pictures [96]. CMRI enables us to examine the impacts of ventricular remodelling that take place as the illness worsens and the escalating severity of the stenosis, which includes individuals having calcific aortic valve disease (CAVD) [97]. As a result, it is a helpful tool for monitoring these individuals [98, 99]. CMRI also contributes information about the size of the aorta when thinking about surgery and offers information about valve morphology, which in some situations enables us to estimate AVA using planimetry. A more functional report based on phase contrast pulse sequences is also possible with CMRI. This kind of sequence involves the simultaneous recording of two different kinds of pictures, one including velocity coding (phase sequences) and another with only anatomical images. These sequences show the stationary tissue as grey, the circulation through the region of interest in the positive direction as white, and the flow through the region of interest in the negative direction as black. It is possible to code velocity in parallel planes or planes perpendicular to the direction of the flow (through the plane) (in the plane). This allows for the measurement of velocity and volume in any vessel at any stage of the cycle and the estimation of the maximal jet speed of the stenosis [100].

#### Computed tomography (CT)

Accurate anatomical pictures of either the aorta root and valve opening are provided by multislice CT. AVA is frequently underestimated when assessed by echocardiography since the LVOT is typically not circular rather eccentric, as seen by the growing adoption of this technique preceding TAVI to quantify annular size [14]. The accuracy of the AVA estimate would be improved by employing CT to measure the size of the LVOT. The advantage of this method is that the calcium load at the valve level may be measured. The Agatston score, which has a strong connection with echocardiographic data and is a key indicator of a poor prognosis and illness progression when high, is employed for this [15]. In order to distinguish between severe and mild AS, recent research [101] proposed a cut-off point (2065 Agatston units for males and 1274 Agatston units for women). This extra metric, in addition to being flow independent, might assist to characterise the severity of the condition in contentious individuals, such as those who have significant AS, low flow, & low gradient. However, further research is required to verify its predictive usefulness.

### Management of individual with DAS and DM

#### Treatment/therapies

**Fig. 6:**
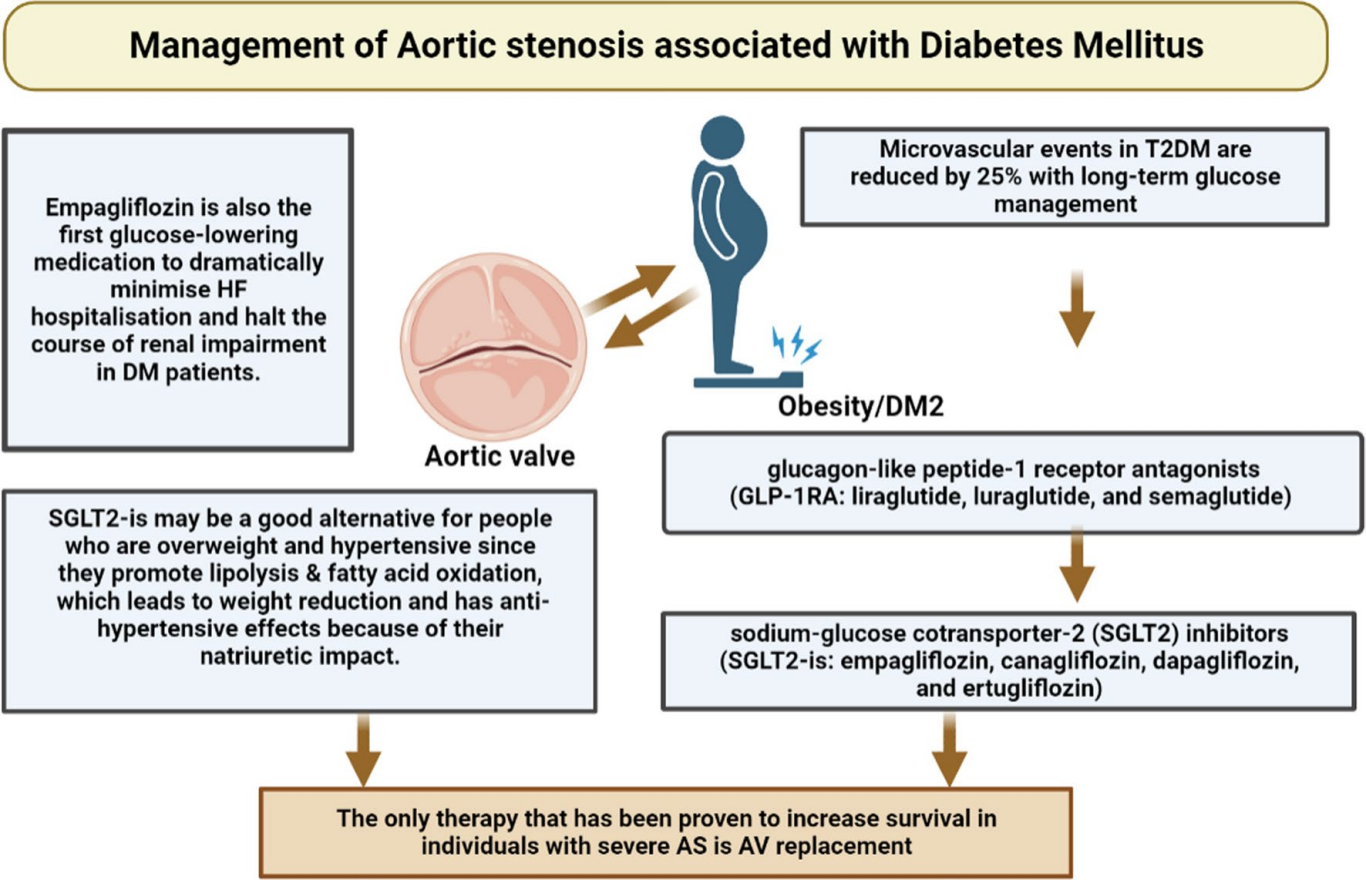
Diagrammatic representation of management of Aortic Stenosis associated with Diabetes Mellitus

It is widely known that poor glycaemic control or cardiovascular events are related. Cardiac Effects Throughout Glycaemic Control in T2DM Patients. Microvascular events in T2DM are reduced by 25% with long-term glucose management, while macrovascular events like myocardial infarction (MI) and stroke are unaffected. For T2DM, a number of medications have been created to provide successful glycaemic control [102]. In fact, to meet HbA1c objectives, current recommendations advise using a mix of glucose-lowering medications, which is typically difficult for doctors to do, especially when treating patients who also have concurrent cardiac disease. In fact, the majority of routinely prescribed anti-diabetic medications are contraindicated in people with heart failure (HF). As a result, there is an urgent requirement for an oral agent that can enhance glycaemic management while also having positive cardiovascular effects. The only clinical trials of interest for cardiovascular purposes are those using glucagon-like peptide-1 receptor antagonists (GLP-1RA: liraglutide, luraglutide, and semaglutide) & sodium-glucose cotransporter-2 (SGLT2) inhibitors (SGLT2-is: empagliflozin, canagliflozin, dapagliflozin, and ertugliflozin). Approximately 90% of the reabsorption of filtered glucose is carried out by SGLT2 proteins [103, 104]. As SGLT2-is activity is independent of cell function, it lowers blood glucose without increasing insulin secretion via decreasing renal tubular glucose reabsorption. As a result, SGLT2-is may be a good alternative for people who are overweight and hypertensive since they promote lipolysis & fatty acid oxidation, which leads to weight reduction and has anti-hypertensive effects because of their natriuretic impact (Fig. 6).

#### SGLT2 inhibitors and cardiovascular outcomes

**Fig. 7:**
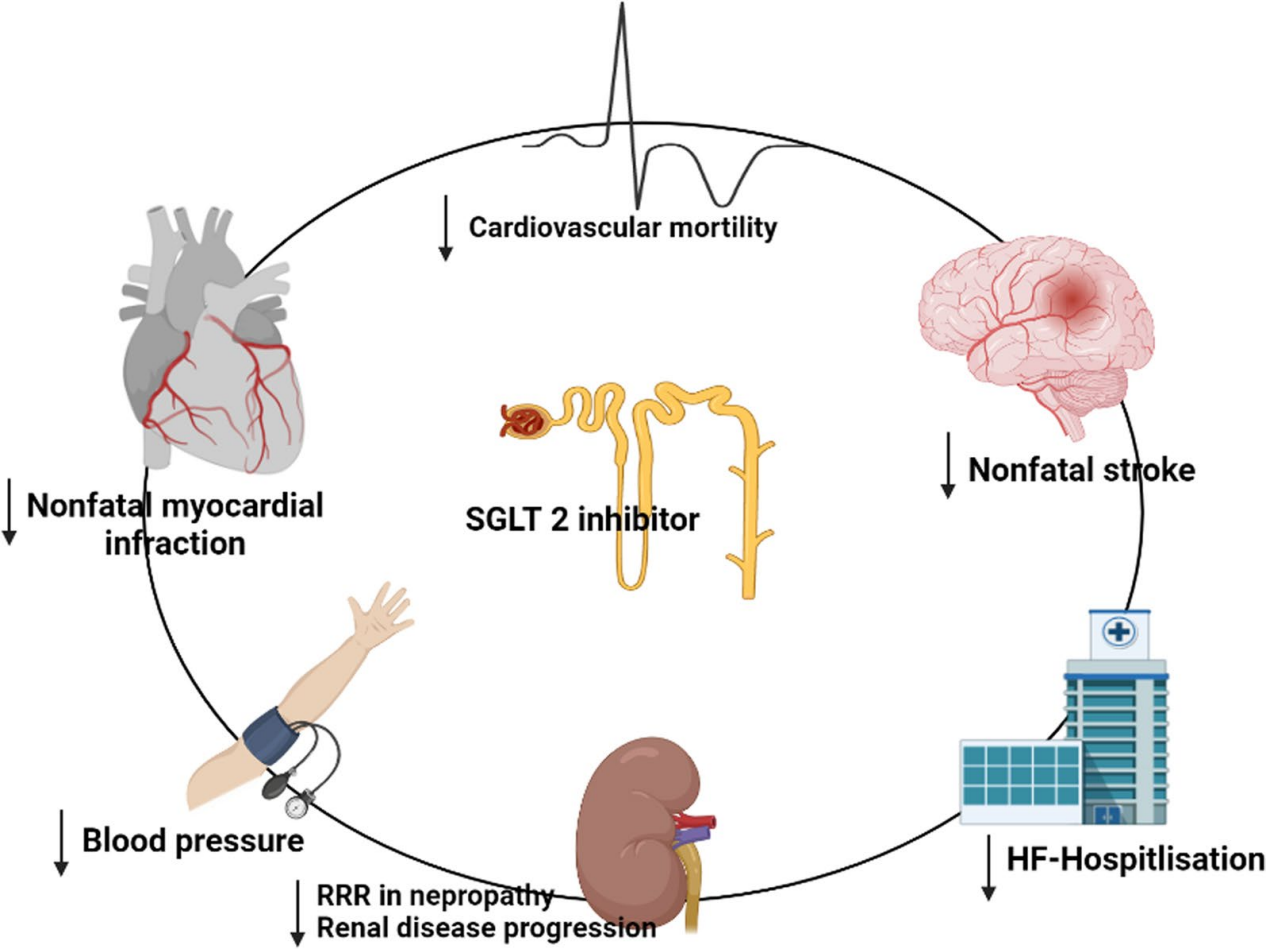
SGLT 2 inhibitor and their cardiovascular outcomes

SGLT2-is influence T2DM patients and improve arterial stiffness by delaying the development of microvascular alterations. A significant therapeutic advance in the management of T2DM patients was suggested by the current EMPAREG OUTCOME study [105]. In addition to receiving conventional medical care, patients with a moderate cardiovascular risk (47% had a history of MI and 25% had a history of stroke) were randomly assigned to receive therapy with empagliflozin or a placebo. Due to the significant cardiac advantages, the experiment was abruptly ended. The key aggregate terminal of cardiovascular mortality, non-fatal MI, and non-fatal stroke was specifically decreased by empagliflozin by 14% (HR 0.86; (0.74–0.99), p = 0.04 for superiority). The patients who took empagliflozin also saw a substantial decrease in the secondary endpoints, including a 38% relative risk reduction (RRR) for cardiovascular mortality, a 32% RRR for death from any cause, and a 35% RRR for hospitalisation due to HF. Empagliflozin is also the first glucose-lowering medication to dramatically minimise HF hospitalisation and halt the course of renal impairment in DM patients. Empagliflozin demonstrated a 39% RRR for incident/worsening nephropathy as well as a 44% RRR for doubling blood creatinine levels in a significant, randomised study [106] with more than 7000 individuals (Fig. 7).

#### Treatment and prognosis of AS in patients with DM: the impact of DM after trans-catheterAV implantation

**Fig. 8:**
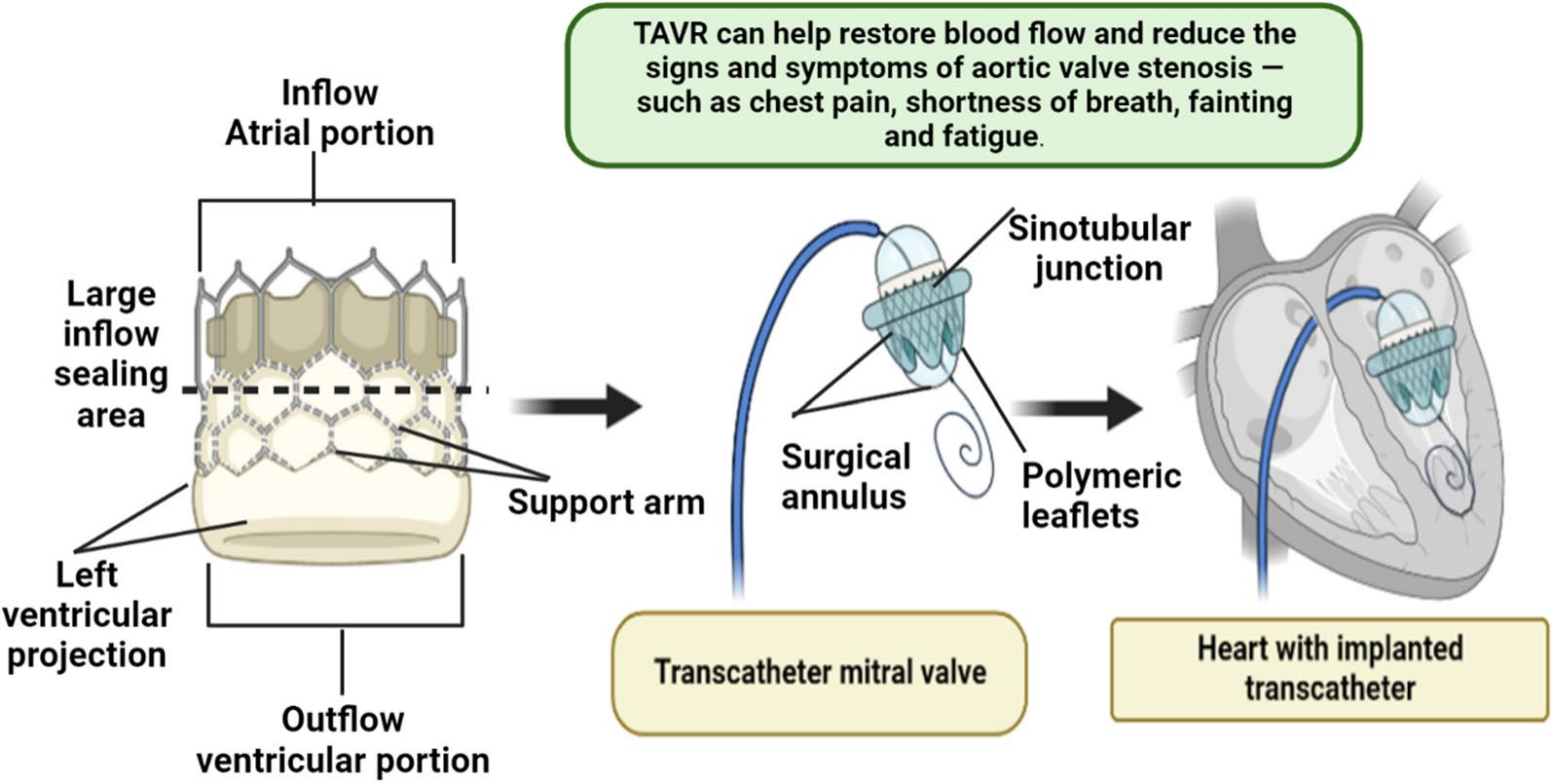
Heart with Implanted Transcatheter

Diabetes patients with severe AS may be treated with anti-diabetic drugs that target the valve or perhaps the myocardium, including oral pills and insulin. Theoretically, targeted medical treatment should stop the evolution of AS, lessen its hemodynamic effects on LV function &remodelling, and enhance clinical outcomes. The survival of AS patients does not appear to be impacted by any of the existing treatments for heart illness or comorbidities, and there is no evidence that they can delay the progression of the disease [107]. The only therapy that has been proven to increase survival in individuals with severe AS is AV replacement, which has a higher post-operative morbidity and death rate in diabetic patients than in non-diabetic patients [108]. Regardless of whether the trans-femoral as well as trans-apical technique was employed, these outcomes remained the same. Additionally, DM hinders the LV mass regression following AV replacement, suggesting that diabetic patients receive less advantages and are more at risk for AV replacement than non-diabetic patients [109, 110]. T2DM patients, however, have reduced intrahospital mortality following AV replacement than non-diabetic patients, according to recent large retrospective research (Fig. 8).

## Conclusions

The cost of treating calcific aortic valve stenosis is rising, but there is no effective pharmaceutical treatment to delay the need for surgery. The aforementioned medicinal therapies have not yet been conclusively demonstrated to reduce disease development or to enhance clinical outcomes, despite some encouraging results.

The information at hand indicates that DM is linked to a higher incidence of AS, which causes AS to proceed more quickly. However, it is unclear how DM affects AS development, particularly in the beginning. Additionally, it was demonstrated that AGE accumulation—which are more significant mediators of accelerated glycation than hyperglycemia and lead to increased oxidative stress and inflammation—means that glycemic management alone is insufficient to avoid DM symptoms. Furthermore, AGEs levels are more accurate indicators of vascular calcification and DM development than HbA1c. Markers linked with HbA1c and fructosamine were seen in AS patients with concurrent type-2 DM valvular inflammation & calcification, supporting the requirement for stringent long-term glycemic management. Large prospective randomised studies should be conducted to validate this observation.

Currently, the results show a link among AS and DM and that DM negatively impacts the QoL and longevity of AS patients. The sole successful treatment, despite ongoing efforts to find new therapeutic modalities, is aortic valve replacement. More research is required to find methods that can slow the advancement of these conditions, enhancing the prognosis and course of people with AS and DM.

## Data Availability

Not applicable.

## Abbreviations

ECM: Extra cellular matrix
CAVD: Calcified aortic valve diseases
TGF: Transforming growth factor
TNF: Tumour necrosis factor
AGII: Angiotensin II
MI: Myocardial infraction
TAVI: Transcatheter aortic valve implantation
VIC: Valve interstitial cell
IL: Interleukin
LDL: Low-density lipoprotein
ACE: Angiotensin-converting enzyme
BMI: Body mass index
VEC: Valvular endothelial cell
